# Creation of Cybrid Cultures Containing mtDNA Mutations m.12315G>A and m.1555G>A, Associated with Atherosclerosis

**DOI:** 10.3390/biom9090499

**Published:** 2019-09-18

**Authors:** Margarita A. Sazonova, Vasily V. Sinyov, Anastasia I. Ryzhkova, Marina D. Sazonova, Zukhra B. Khasanova, Tatiana P. Shkurat, Vasily P. Karagodin, Alexander N. Orekhov, Igor A. Sobenin

**Affiliations:** 1 Laboratory of Angiopathology, Institute of General Pathology and Pathophysiology, 125315 Moscow, Russia; ryzhkovaai@gmail.com (A.I.R.); marinasazon1990@gmail.com (M.D.S.); vpka@mail.ru (V.P.K.); a.h.opexob@gmail.com (A.N.O.); igor.sobenin@gmail.com (I.A.S.); 2 Laboratory of Medical Genetics, National Medical Research Center of Cardiology, 121552 Moscow, Russia; centaureaceanus@mail.ru (V.V.S.); zukhra@yandex.ru (Z.B.K.); 3 Plekhanov Russian University of Economics, 117997 Moscow, Russia; 4 Department of Genetics, Southern Federal University, 344006 Rostov-on-Don, Russia; tshkurat@yandex.ru; 5 Institute for Atherosclerosis Research, Skolkovo Innovation Centre, 121609 Moscow, Russia

**Keywords:** cytoplasmatic hybrids, mitochondrial genome, mutation, gene, cybrids, mtDNA, therapy, cybrid model

## Abstract

In the present work, a pilot creation of four cybrid cultures with high heteroplasmy level was performed using mitochondrial genome mutations m.12315G>A and m.1555G>A. According to data of our preliminary studies, the threshold heteroplasmy level of mutation m.12315G>A is associated with atherosclerosis. At the same time, for a mutation m.1555G>A, such a heteroplasmy level is associated with the absence of atherosclerosis. Cybrid cultures were created by fusion of rho0-cells and mitochondria from platelets with a high heteroplasmy level of the investigated mutations. To create rho0-cells, THP-1 culture of monocytic origin was taken. According to the results of the study, two cybrid cell lines containing mutation m.12315G>A with the heteroplasmy level above the threshold value (25% and 44%, respectively) were obtained. In addition, two cybrid cell lines containing mutation m.1555G>A with a high heteroplasmy level (24%) were obtained. Cybrid cultures with mtDNA mutation m.12315G>A can be used to model both the occurrence and development of atherosclerosis in cells and the titration of drug therapy for patients with atherosclerosis. With the help of cybrid cultures containing single nucleotide replacement of mitochondrial genome m.1555G>A, it is possible to develop approaches to the gene therapy of atherosclerosis.

## 1. Introduction

At present, cybrid cell cultures are the most promising models for studying pathological processes in cells in various diseases. In particular, they may be used for studying the impact of mtDNA variants on the occurrence of mitochondrial dysfunction and different pathologies in humans [1,2,3,4,5].

One of the relevant goals of medicine and public health is the creation of cellular models containing pathogenic mutations for the development of drug therapy of various pathologies. For example, such cybrid models were developed for the titration of drug therapy for Parkinson’s disease, MELAS syndrome, Leber hereditary optic atrophy, Alzheimer’s disease, HIV, human herpes virus type 8, and Leigh syndrome [1,2,3,4,5]. However, cybrid cell models have not been created before to study the pathogenesis of atherosclerosis.

The advanced field of molecular medicine encompasses the creation of cybrid cell cultures containing mutations with an anti-pathological effect. These cultures are necessary for gene therapy of human diseases.

Atherosclerosis is one of the most severe diseases of the 21st century [6,7,8,9,10]. Every year, a large number of people die of it [11,12,13,14,15]. According to the literature, one of the causes of atherosclerosis is mitochondrial dysfunction [16,17,18,19,20]. In particular, the association of atherosclerosis with mitophagy was detected [16,17,18]. Signal pathways that link mitochondrial dysfunction with shortening of telomeres, oxidative stress, and atherosclerosis were found [18,19,20,21]. Moreover, it was found that metabolic disorders can lead to a defect in the mitochondrial membrane, leading to mitochondrial dysfunction. This can cause oxidative stress and atherosclerotic lesions in humans [22,23,24].

However, no attempts have yet been made to create cybrid cell models containing atherogenic and antiatherogenic mitochondrial genome mutations.

In the present work, a pilot creation of four cybrid cultures with a high heteroplasmy level was performed using mitochondrial genome mutations m.12315G>A and m.1555G>A. Mutation m.12315G>A is localized in gene *MT-TL2*. As a result of this mutation, transport RNA-Leucine dysfunction (CUN recognition codon) can occur, with a subsequent decrease in the level of synthesis of mitochondrial proteins on the ribosome. Single nucleotide substitution m.1555G>A is localized in gene *MT-RNR1*. As a result of mutation m.1555G>A, the 12S subunit of ribosomal RNA can stabilize, leading to an acceleration of the protein chains synthesis on the mitochondrial ribosome.

## 2. Results and Discussion

In the literature sources, we have not found methods for creating rho0-cells based on monocyte-derived culture THP-1. Therefore, in the present research, such conditions are made for the first time.

The algorithm for creating rho0-culture THP-1 is described in the “Materials and Methods” section. It should be noted that for the creation of morphologically homogeneous and resistant to repeated passaging of the cybrid lines, in general, established cell lines are used, i.e., those cells which have passed the stage of dedifferentiation. In our case, the total period of obtaining rho0-culture THP-1 was 18 weeks. The absence of mitochondria in the culture was confirmed by the analysis of the number of mitochondrial genome copies in rho0 culture using the real-time PCR method. To do this, a set of reagents for real-time PCR in the presence of SYBR Green I (Sintol, Russia) was used (Table 1). The native cells culture THP-1 was a control sample. If the number of mitochondrial genome copies in the culture was very small or there was no mtDNA at all, unlike native THP-1, it was considered that the rho0-culture of THP-1 was created.

Cybrid cultures were obtained by PEG-fusion of rho0 cells and mitochondria from platelets of patients. The criterion for selection of platelet donors was the threshold heteroplasmy level of mtDNA mutations m.12315G>A and m.1555G>A. According to our preliminary studies, mitochondrial genome m.12315G>A mutation was associated with atherosclerotic lesions in the intima of human arteries [25,26,27,28,29]. At the same time, mtDNA mutation m.1555G>A had a protective effect in atherosclerosis, i.e., it was anti-atherogenic [25,26,27,28,29].

A comparison of the number of copies of the mitochondrial genome in the resulting cybrid cultures and the rho0 culture of THP-1 was made using a set of real-time PCR reagents in the presence of SYBR Green I (Syntol, Russia). The native culture of THP-1 cells was a control culture. If the number of mitochondrial genome copies in cybrid cultures slightly differed from the number of copies of mtDNA in native THP-1 cell culture, but was significantly larger than in rho0 culture, it was considered that cybrid cultures were created.

In the present study, we have obtained two cybrid cultures for each of the studied mutations. Two cybrid cultures were obtained for mutation m.12315G>A. In one cybrid culture with mutation m.12315G>A, the heteroplasmy level of m.12315G>A was 44%. In another cybrid culture with mutation m.12315G>A, the heteroplasmy level of m.12315G>A was 25%.

The heteroplasmy level of mitochondrial genome mutation m.12315G>A in these 2 cybrid cell cultures exceeded the threshold value detected for these mutations in patients with atherosclerotic plaques (7.5%) and thickened intima-medial layer of the carotid arteries (10.5%) [28]. In the first cybrid line, the heteroplasmy level of mutation m.12315G>A was 25%; and in the second it was 44%. Patients who were donors of platelets for these cybrid cultures had a thickened intima-medial layer of carotid arteries. As a comparison, a cybrid culture with low heteroplasmy level of mutation m.12315G>Awas taken. A patient, who was a donor of platelets for this cybrid culture had normal intima-media.

In addition, two more cybrid cultures were obtained for mutation m.1555G>A. In both cultures, the heteroplasmy level of m.1555G>A was 24%.

The heteroplasmy level m.1555G>A was above the threshold value detected for this mutation in patients with atherosclerotic plaques (17.5%) and thickened intima-medial layer of carotid arteries (19.5%) [28].

Patients, who were donors of platelets for this cybrid cultures, had a normal intima-medial layer of carotid arteries. As a comparison, a cybrid culture with a low heteroplasmy level of mutation m.1555G>A was taken. A patient who was a donor of platelets for this cybrid culture had a thickened intima-medial layer of carotid arteries. A paired comparison of two cybrid cell lines with a high and a low heteroplasmy level for the same mutation can help in investigating the molecular–cellular mechanisms of mitochondrial dysfunction in atherosclerosis and cardiovascular diseases.

The created cybrid cultures contain mutations localized in the coding region of the mitochondrial genome. Mutation m.12315G>A is localized in gene *MT-TL2*. As a result of this mutation, transport RNA-Leucine dysfunction (CUN recognition codon) can occur, with a subsequent decrease in the level of synthesis of mitochondrial proteins on the ribosome. Single nucleotide substitution m.1555G>A is localized in gene *MT-RNR1*. As a result of mutation m.1555G>A, the 12S subunit of ribosomal RNA can stabilize, leading to an accelerated synthesis of protein chains on the mitochondrial ribosome.

The obtained cybrid cell lines can serve as models for studying the molecular–cellular mechanisms of mitochondrial dysfunction in atherosclerosis and cardiovascular diseases. Cybrid cultures with mutation mtDNA m.12315G>A can be used both to model the occurrence and development of atherosclerosis in cells and for the titration of drug therapy for patients with atherosclerosis. With the help of cybrid cultures containing single nucleotide replacement of the mitochondrial genome m.1555G>A, it is possible to develop approaches to gene therapy of atherosclerosis.

## 3. Materials and Methods

### 3.1. Creation of rho0 Cell Cultures

To obtain rho0-human cells, the method of M. King and G. Attardi was used [30].

The algorithm for creating rho0-human cells is as follows:

1. At the first stage, the possibility of creating rho0-cells was tested. For this purpose, a normal culture of monocytic origin, THP-1, was cultured in a growth medium in two variants:

(1) With the addition of uridine and ethidium bromide;
(2) With the addition of ethidium bromide only.

**Figure 1:**
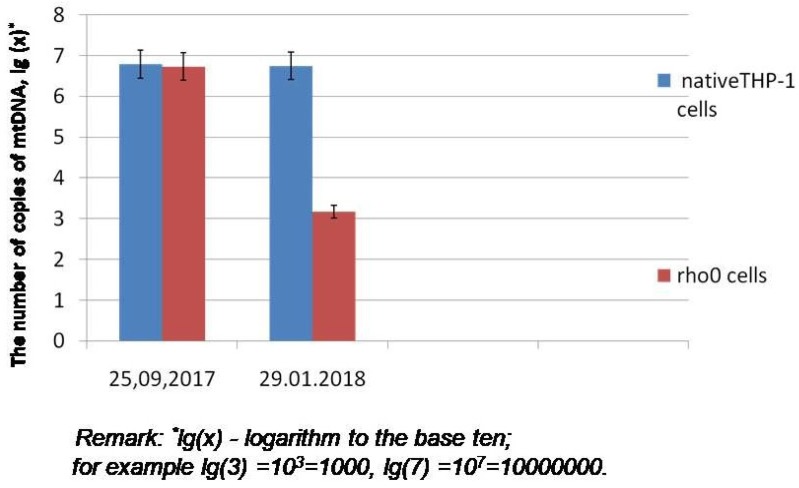
Number of copies of mtDNA in the culture of THP-1 cells (reference matrix-mutation m.12315G>A).

The growth medium contained pyruvate and glucose at the concentration indicated for the complete DMEM medium. The necessary conditions for the cultivation of THP-1 for the creation of rho0-cells were determined:

(1) Time of cultivation;
(2) Concentration of additives used in the medium.

2. At the second stage, the rho0 cell line was created. THP-1 was placed in a growth medium with the addition of uridine and ethidium bromide. With the use of ethidium bromide, the mitochondrial genome was blocked, and complete mitochondrial dysfunction occurred. Afterwards the cultured cell line was placed on medium with only uridine (but without ethidium bromide). In the process of culturing on this medium, the cell line was to lose all non-functioning mitochondria and to become an mtDNA-less cell culture (rho0). It should be noted that it took us 18 weeks to create a rho0 culture of monocytic origin. Thirty-six passages were conducted (two passages per week). If a stable, rho0 cell line was obtained using the culture conditions described above, then an analysis was performed to confirm that the mitochondria in the cell line were absent. To achieve this aim, the analysis of the number of mitochondrial genome copies in rho0 culture was carried out (Figure 1).

In the process of creating rho0 cells and cybrid cultures, the following solutions and media were used (Table 1).

### 3.2. Isolation of Platelets from Blood

Cybrid cells were obtained by fusing rho0-cells with platelets. In this case, platelets were donors of mitochondria.

The work was conducted in accordance with the Declaration of Helsinki. The study protocol has been approved by the Ethics Community of Institute of General Pathology and Pathophysiology, and all donors of the blood platelets gave a written informed consent upon enrollment.

Platelets were isolated from whole blood by ficoll-urografin density gradient centrifugation.

1. Preparation of blood before the isolation of platelets.

To the blood obtained from donors of platelets (patients with atherosclerosis), 10X sodium citrate solution was added in physiological solution in the ratio 1:1 (Table 1). The resulting mixture was centrifuged for 20 min at 200× *g* at a temperature of 12 °C.

2. Isolation of platelets.

Three-quarters of the supernatant (plasma) was recovered and centrifuged for 20 min at 1500× *g* at a temperature of 15 °C. The supernatant was then taken away. To the remaining precipitate, 11 mL of physiological solution was added.

3. Cryogenic storage of platelets.

To 11 mL of the platelet suspension in physiological solution, 1.5 mL of sterile DMSO and 3 mL of FBS was added. Cryovials were placed in a test-tube holder for controlled freezing of the cells (1 °C/min) for 8 h at a temperature of −80 °C. Afterwards, the samples were stored in liquid nitrogen.

### 3.3. Creation of Cybrid Cultures

The penetration of mitochondria into rho0-cells can occur only through pores. They are formed in cytoplasmic membranes of cells as a result of exposure to some chemical or physical factors. It should be noted that in the literature there is no information on the creation of cybrid cell cultures based on culture of monocytic origin THP-1. Therefore, in the present work, these cybrid cultures were created for the first time.

The technique of “PEG-fusion” was used, which allows the creation of cytoplasmic hybrids by fusing rho0-cells with platelets. In this case, platelets were donors of mitochondria. It should be emphasized that the use of platelets greatly simplifies the protocol for the production of cybrid lines, since platelets contain only mitochondrial genome. Nuclear genome is absent in them.

1. Preparation of cells before fusion.

If the platelets were frozen, they were first subjected to defrosting and removal of the cryoprotectant from the cells. For this purpose, a suspension with platelets was added into 14 mL of physiological solution heated at 37 °C (Table 1). A cryovial with platelets was preheated in a water bath at 37 °C. Further, it was centrifuged for 15 min at 1500× *g* at a temperature of 15 °C. The supernatant was then taken away.

2. Preparation of rho0-cells.

The cell suspension was centrifuged for 5 min at 180× *g* at 25 °C. Rho0 cells were resuspended in the DMEM [-Ca^2+^] medium at a concentration of 5 × 10^5^ cells/mL.

3. Fusion of cells.

Rho0 cells, prepared at stage 2, were added to the platelet precipitate, prepared at stage 1. The cell mixture was centrifuged for 10 min at 180× *g* at 25 °C. The supernatant was taken away. Then, 100 μL of 42% PEG was added to the platelet precipitate with rho0 cells, and afterwards, this mixture was resuspended. In 1 min, it was resuspended for 30 s.

The resulting cell suspension was cultured in a growth medium in a CO_2_ incubator at a temperature of 37 °C.

### 3.4. Verification of Mitochondrial Genome Copies Number in rho0 (mtDNA-less) and Cybrid Cell Cultures

In the created rho0 and cybrid cell cultures, the number of mitochondrial genome copies was detected. With the help of this parameter, either “mtDNA-lessness” (rho0-cells) or the presence of mitochondria (cybrids) was confirmed (Figure 1).

The number of copies of the mitochondrial genome was detected using synthetic matrices based on the mitochondrial DNA region at concentrations of 10^3^, 10^4^, 10^5^, and 10^6^. Based on the Ct-value data of the matrices, a calibration curve was constructed which was used to detect the number of mtDNA copies in the investigated cell culture samples.

The heteroplasmy level of mutations 12315G>A and 1555A> G in rho0 cells was extremely low. This was connected with the fact that the number of copies of mitochondrial genome in rho0 cells was very small compared to the native THP-1 culture. For example, in the studied sample of total DNA (30 ng/μL) from the rho0 culture, there were approximately 10^3^ copies of mtDNA. At the same time, the sample from the native THP-1 culture contained more than 10^6^ copies of mitochondrial genome (Figure 1). After obtaining cybrid cultures containing the studied mutations, the heteroplasmy level of these single-nucleotide substitutions in cybrid cells was approximately the same as in the platelets of donor patients.

### 3.5. Determination of the Heteroplasmy Level of Mitochondrial Genome Mutations m.12315G>A and m.1555G>A

With the help of rho0-cell lines, cybrid cell cultures with the high heteroplasmy level of mitochondrial genome mutations m.12315G>A and m.1555G>A were created. The heteroplasmy level of mtDNA mutations m.12315G>A and m.1555G>A was detected with the use of the original method of quantitative assessment of the mutant allele in mitochondrial genome, developed by M.A. Sazonova and colleagues on the basis of pyrosequencing technology [25,26,27,28,29].

The PCR fragments of the DNA of platelet donors, containing the region of the investigated mutations, were subjected to pyrosequencing. Based on the developed formula, the heteroplasmy level of these mutations was detected [25,26,27,28,29].

The following PCR primers were used [25,26,27,31,32]:

1. For m.12315G>A

R: TTACTTTTATTTGGAGTTGCAC(12337-12317);

F: bio-CTCATGCCCCCATGTCTAA(12230-12249).

2. For m.1555A>G

R: bio-GTAAGGTGGAGTGGGTTTGGG(1704-1684);

F: TAGGTCAAGGTGTAGCCCATGAGGTGGCAA(1326-1355).

A standard buffer with ammonium sulfate was used for PCR. The concentration of magnesium chloride in the buffer was 2.5 mM. The size of the amplification for the mutation m.12315G>A was 108 bp, and for m.1555A>G was 379 bp [25,26,27,31,32].

The following primers for pyrosequencing were used [25,26,27,31,32]:

1. For m.12315G>A

TTTGGAGTTGCAC (12328-12316).

2. For m.1555A>G

ACGCATTTATATAGAGGA (1537-1554).

## 4. Conclusions

In the present research work, a pilot creation of four cybrid cultures with high heteroplasmy level was performed using mitochondrial genome mutations m.12315G>A and m.1555G>A. According to the data of our preliminary studies, the threshold heteroplasmy level of mutation m.12315G>A is associated with atherosclerosis. At the same time, for a mutation m.1555G>A, such a heteroplasmy level is associated with the absence of atherosclerosis.

The obtained cybrid cell lines can serve as models for studying the molecular–cellular mechanisms of mitochondrial dysfunction in atherosclerosis and cardiovascular diseases. Cybrid cultures with mutation mtDNA m.12315G>A can be used both to model the occurrence and development of atherosclerosis in cells and for the titration of drug therapy for patients with atherosclerosis. With the help of cybrid cultures containing single nucleotide replacement of mitochondrial genome m.1555G>A, it is possible to develop approaches to gene therapy of atherosclerosis.

## Figures and Tables

**Table 1:** Solutions and media.

**Growth Medium**		
***Reagent***	*Concentration*	*Company*
RPMI-1640	-	Gibco
l-glutamine	300 mg/L	Paneco
2-mercaptoethanol	2 × 10^−5^ M	Acros
Foetal bovine serum (FBS)	10% of the total volume	HyClone
Penicillin-streptomycin, 100x solution	penicillin—50 U/mL, streptomycin—50 µg/ml	Paneco
Dextroglucose	2500 mg/L	Amresco
Sodium pyruvate	110 µg/L	Paneco
**10X Sodium Citrate in Physiological Solution**		
***Reagents***	*Concentration*	*Company*
Physiological solution	0.15 M NaCl	Paneco
Sodium citrate (Na_3_C_6_H_5_O_7_·2H_2_O) trisodium salt, dihydrate)	0.10 M	Amresco
*Polyethylene glycol solution*		
Polyethylene glycol 1500	42% of the total volume	Acros
Dimethylsulphoxide(DMSO)	2 mL	Amresco
DMEM [-Ca^2+^]	9.5 mL	Gibco
**Ready-to-Use Solutions and Media**		
***Reagents***	*Concentration*	*Company*
Uridine	50 mg/mL solution	Sigma-Aldrich
Ethidium bromide	1% solution	AppliChem
Ficoll-urografin	density 1.077	Paneco
DMSO		Amresco
Physiological solution	0.15 M NaCl	Paneco
DMEM [-Ca^2+^]		Gibco
Reagent kit for conducting real-time PCR in the presence of SYBR Green I		Synthol

## Abbreviations

DNA	Deoxyribonucleic acid
mtDNA	mitochondrial DNA
DMEM	Dulbecco modified Eagle medium
HIV	Human immunodeficiency virus
FBS	Foetal bovine serum
MELAS	Mitochondrial encephalomyopathy, lactic acidosis, and stroke-like episodes
RNA	Ribonucleic acid
DMSO	dimethylsulphoxide
PEG	polyethylene glycol
